# Ni-Ru/CeO_2_ Catalytic Hydrothermal Upgrading of Water-Insoluble Biocrude from Algae Hydrothermal Liquefaction

**DOI:** 10.1155/2018/8376127

**Published:** 2018-05-08

**Authors:** Donghai Xu, Shuwei Guo, Liang Liu, Hui Hua, Yang Guo, Shuzhong Wang, Zefeng Jing

**Affiliations:** Key Laboratory of Thermo-Fluid Science & Engineering, Ministry of Education, School of Energy and Power Engineering, Xi'an Jiaotong University, Xi'an, Shaanxi Province 710049, China

## Abstract

Hydrothermal liquefaction (HTL) of algae is a promising crude bio-oil (biocrude) production technology, which can convert wet algae into water-insoluble biocrude and other coproducts. In this work, algae HTL at 350°C and 20 min was conducted to obtain water-insoluble biocrude (B_1_), which was then hydrothermally upgraded at 450°C, 60 min, or with added H_2_ and/or homemade catalyst (i.e., Ni-Ru/CeO_2_ or Ni/CeO_2_) for the first time. The characteristics (e.g., yield, elemental component, energy recovery, and molecular and functional group compositions) of upgraded water-insoluble biocrude (B_2_) as well as light biocrude thereof were analyzed comprehensively. The results show that Ni-Ru/CeO_2_+H_2_ led to the highest yield and HHV (higher heating value), the best elemental compositions quality of B_2_, and the largest fraction and the best light of light biocrude in B_2_. Ni-Ru/CeO_2_+H_2_ had good catalytic desulfurization effect and could transform high-molecular-weight compounds into low-molecular-weight compounds in B_1_ upgrading. At the condition above, 46.2% of chemical energy in the initial algae could be recovered by B_2_, while average 54.9% of chemical energy in B_2_ was distributed in its light biocrude (hexane-soluble) portion. On the whole, Ni-Ru/CeO_2_+H_2_ can be considered as the optimal additive in all tested cases.

## 1. Introduction

Hydrothermal liquefaction (HTL) of algae for crude bio-oil (biocrude) production has increasingly drawn much attention due to its benign raw material properties, high bio-oil yield, avoidance of drying step, and so on. It can chemically convert high moisture algae into water-insoluble biocrude and coproducts (i.e., gases, aqueous phase, and solids) in hot compressed water. However, the obtained biocrude does not meet biodiesel standards in some aspects such as heteroatoms content and heating value, so it requires further biorefinery via proper approaches. An effective method is hydrothermal upgrading, in which biocrude reacts in water, either thermally or with added H_2_ and/or catalyst to remove heteroatoms and improve heating value, and so on [1].

Typically, after algae HTL of 1–4 h at around 350°C, total biocrude (both water-insoluble and water-soluble biocrudes) is collected by organic solvent (e.g., dichloromethane) extraction from all postreaction products and followed hydrothermal upgrading with 2–6 h at a supercritical temperature [2–7]. Notably, the water-insoluble biocrude can spontaneously separate from aqueous phase by gravity upon cooling after algae HTL and commonly accounts for more than 85 wt% of the total biocrude and has remarkably distinct characteristics in comparison to the water-soluble biocrude extracted from the aqueous phase by organic solvent [8, 9]. Due to pollution and cost restrictions of extraction solvent, it is more feasible to adopt solvent-free method to collect and upgrade the water-insoluble biocrude (as the majority of the total biocrude) in actual industrial production. On the other hand, it is convenient and economic to directly use the aqueous phase instead of water in hydrothermal upgrading, because it is easy to separate part of the aqueous phase after algae HTL and to use the rest for following hydrothermal upgrading. However, relevant investigation about water-insoluble biocrude upgrading with the aqueous phase and catalyst is very scarce now, except for our latest research about catalytic hydrothermal upgrading of water-insoluble biocrude in supercritical water at 400°C, 60 min with commercial catalysts (i.e., Mo_2_C, Ru/C, and Pt/C ) [1].

As an extension, this work conducted 20 min of algae HTL at 350°C to obtain water-insoluble biocrude and then upgraded it at the higher temperature of 450°C, 60 min with added H_2_ and/or Ni-Ru bimetallic catalyst homemade for the first time. The yields, elemental components, energy recoveries, and molecular and functional group compositions of the upgraded water-insoluble biocrudes at various conditions were analyzed systematically. The characteristics concerning the light biocrude in the upgraded biocrude and gaseous products were examined as well. This information is valuable for optimizing processes design and operation parameters of the hydrothermal upgrading of the water-insoluble biocrude from algae HTL.

## 2. Experimental Section

### 2.1. Experimental Procedures

The adopted algae (*Nannochloropsis* sp.) slurry had 30.5 ± 2.5 wt% of biomass content, which was determined by drying three separate samples at 65°C in an oven for 48 h. The dry basis algae (about 23.30 MJ/kg) contained approximately 52.85, 7.43, 8.78, and 0.62 wt% of C, H, N, and S contents, respectively. High purity solvents (i.e., dichloromethane (DCM), n-hexane, and deuterated chloroform (CDCl_3_)) were obtained from commercial sources. High purity (≥99.999%) hydrogen and helium were purchased from Baoguang Gas Co., Ltd. Several 4.1 ml mini-batch reactors were assembled for the HTL experiments by using 1/2-in. 316 stainless steel Swagelok port connectors and caps. Subsequent biocrude upgrading experiments were conducted in the same reactors, but upgrading reactions with added H_2_ or with gas analysis were implemented by additionally equipping each reactor with a length of stainless steel tubing and a high-pressure gas valve.

First of all, one set of experiments were carried out to obtain water-insoluble biocrude from algae HTL at 350°C, 20 min, and 14.1 wt% of algae loading in the reactor (corresponding to 1.2354 g algae slurry and 1.3990 g deionized water). After being sealed, the reactor was placed in a Techne fluidized sand bath (Model SBL-2) preheated up to 350°C for the desired residence time (20 min). Herein, the corresponding reaction pressure was about 16.5 MPa and the reactor heating-up time was 2-3 min. The postreaction reactor was removed from the sand bath and quenched in an ambient-temperature water bath for 15 min and then equilibrated at room temperature for at least 1 h before products collection and analysis. The reactor was kept in a vertical position as it was cooled so that little biocrude would adhere to the inner surface of the reactor top cap. We then opened the reactor and removed 1.875 g aqueous phase via a pipette. The water-insoluble biocrude (about 0.1505 g) from algae HTL (with a little solids residue) and the aqueous phase residue (around 0.3750 g) in the reactor were never in contact with DCM and were used in situ for the following upgrading experiments. 0.0225 g catalyst (if necessary, about 15 wt% of B_1_ mass) was loaded into the reactor, and then the reactor was vacuumed and filled either with helium (10 KPa) to eliminate the effect of reactor inside air in the test without H_2_ addition or with H_2_ to 2.0 MPa to check the effect of H_2_ addition. The upgrading experiments were carried out in the sand bath at 450°C, 60 min under different conditions such as catalyst and/or H_2_ addition, and the corresponding reaction pressure was approximately 22.5 MPa (based on steam tables). We then removed the reactor from the sand bath and cooled it and followed the previously described method 2 procedure [8] for products collection. In this procedure, the contact between the aqueous phase and DCM is avoided by removing the aqueous product via a pipette, prior to the recovery of the water-insoluble biocrude from the reactor. Eventually, water-insoluble biocrude (B_2_) was obtained from B_1_ upgrading products, and light biocrude in B_2_ (B_2_^L^) could be further gained by n-hexane extraction.

The mainly used catalysts were 10 wt%Ni0.1Ru/CeO_2_ and 10 wt%Ni/CeO_2_, which were prepared in house and detailed procedures had been described by our previous report [10]. Herein, 10 wt% represents the mass percentage of active metal (Ni+Ru or Ni) in the catalyst, and 0.1 is the mass ratio of Ru/Ni. For simplification, Ni-Ru/CeO_2_, Ni/CeO_2_, and “none” were adopted to separately represent 10 wt%Ni0.1Ru/CeO_2_, 10 wt%Ni/CeO_2_, and the case without catalyst and H_2_ in this work. Herein, the cases of Ni/CeO_2_ and “none” were tested for comparison with that of Ni-Ru/CeO_2_.

### 2.2. Analysis Methods

Elemental compositions (i.e., C, H, N, and S) of dry basis algae and all biocrude samples were determined by a cube CHNS elemental analyzer (Elementar Vario EL) with uncertainties of <3% of the reported value. Compound components of biocrude were identified via a gas chromatography-mass spectrometer (GC-MS, Agilent Technologies 6890N) equipped with an autosampler, an autoinjector, a mass spectrometric detector, and an Agilent HP-5 capillary column (50 m × 200 *μ*m × 0.33 *μ*m). Nuclear magnetic resonance (NMR) and Fourier transform infrared (FT-IR) spectroscopic analyses of biocrude were conducted to characterize functional group compositions. Detailed information on these instruments and procedures was introduced in our previous research [8]. Gaseous products were identified and quantified by an Agilent Technologies model 6890N gas chromatograph equipped with a thermal conductivity detector, following the method reported previously [11].

Biocrude yield and energy recovery were defined as follows:

(1) Biocrude yield=Mass of biocrudeMass of dry basis algae loaded into the reactor×100%Energy recovery=HHV of biocrude×the biocrude yieldHHV of dry basis algae×100%

The higher heating value (HHV) of dry basis algae or biocrude was estimated by the following Dulong formula:

(2) $$\begin{array}{c} \text{HHV}\left(\;\text{MJ}/\text{kg}\;\right)=0.338C+1.428\left(\;H-\frac{O}{8}\;\right)+0.095S, \end{array}$$

where C, H, O, and S are the wt% composition of each element in the material. The O content was calculated by the differences from the C, H, N, and S values and reserving 1 wt% for other elements not assessed.

Three independent experiments were carried out at the same conditions to determine the uncertainties of experimental data. The results reported herein are mean values and their uncertainties are the sample standard deviations.

## 3. Results and Discussion

All experiments about algae HTL and biocrude upgrading produced water-insoluble biocrude, gases, aqueous phase, and solids, and total mass balance exceeded 91.0 wt% in products collection.

### 3.1. Biocrude Yield

**Figure 1:**
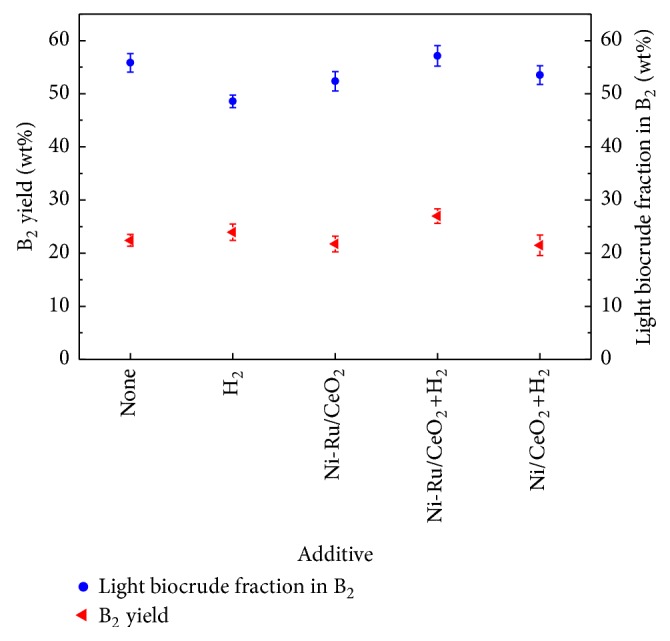
B_2_ yield and light biocrude fraction in B_2_ after B_1_ (derived from algae HTL at 350°C, 20 min) upgrading at 450°C, 60 min with or without H_2_ and/or catalyst.

Algae HTL at 350°C, 20 min, and 14.1 wt% of algae loading led to 37.5 ± 0.21 wt% of B_1_ yield.  Figure 1 shows B_2_ properties after B_1_ upgrading at 450°C, 60 min, with or without H_2_ and/or catalyst. It can be found that Ni-Ru/CeO_2_+H_2_ led to the highest B_2_ yield (27.0 wt%, corresponding to about 72.0 wt% of B_1_ yield), likely due to catalytic hydrogenation effect during B_1_ upgrading. The much lower B_2_ yield (21.72 wt%) appeared at the Ni-Ru/CeO_2_ condition, and this might be attributed to a large amount of gases formation promoted by the catalyst in supercritical water [10]. Based on the B_2_ yield (23.94 wt%) in the case of H_2_ addition, there seemingly was synergetic effect between H_2_ and Ni-Ru/CeO_2_ on B_2_ yield improvement because their coexistence probably inhibits gases formations. Light biocrude fraction in biocrude, which was defined as the measured mass of light biocrude (hexane-soluble) divided by the biocrude mass, is a proxy for biocrude quality because these hexane-soluble compounds are less polar and of moderate boiling point and thus more desirable for fuel use, compared with heavy biocrude (hexane-insoluble) compounds [9]. As indicated in Figure 1, Ni-Ru/CeO_2_+H_2_ could obviously increase the light biocrude fraction in B_2_ and led to the highest value (57.14 wt%), meaning the remarkable improvement of B_2_ quality. Herein, similar synergetic effect between H_2_ and Ni-Ru/CeO_2_ on the promotion of the light biocrude fraction in B_2_ was found as well, probably due to the conversion of hexane-insoluble biocrude to hexane-soluble biocrude in hydrothermal upgrading at the Ni-Ru/CeO_2_+H_2_ condition. Thereby, we could adopt the Ni-Ru/CeO_2_ catalyst together with H_2_ to regulate the properties of the upgraded water-insoluble biocrude. It is worthy noticing that the upgrading without H_2_ and catalyst (i.e., none) rendered considerably high B_2_ yield and light biocrude fraction in B_2_, so it may be considered as a pre-upgrading process of B_1_ in actual large scale production.

### 3.2. Elemental Composition and HHV

Table 1 indicates elemental compositions and HHVs of B_2_ and B_2_^L^ after B_1_ upgrading under different conditions. It can be observed that hydrothermal upgrading could increase the C content and simultaneously reduce S and O contents. Deoxygenation can be realized by forming CO_2_, H_2_O, and CO through complex reactions such as decarboxylation and dehydration [12, 13]. Note that the Ni-Ru/CeO_2_ catalyst had the best catalytic desulfurization effect and Ni-Ru/CeO_2_+H_2_ was also much better for sulfur removal. This has good agreement with the findings that the S content in bio-oil by Ni/SiO_2_-Al_2_O_3_ catalytic upgrading is below detection limit [14] and Ru/C has good desulfurization performance [15]. Ni-Ru/CeO_2_+H_2_ rendered the highest C content, the largest HHV, the lowest O content, and the second lowest N+S content, so totally exhibiting the best catalytic upgrading effect in all tested cases. Consistently, H_2_ addition together with catalyst is desirable for hydrogenation and hydrodeoxygenation behaviors in biocrude upgrading [3, 4, 6]. Apparently, based on the higher C, H contents and HHV but lower heteroatoms (i.e., N, O, and S) contents, each B_2_^L^ had better quality than its corresponding B_2_. Thus, the tested catalysts (i.e., Ni-Ru/CeO_2_ and Ni/CeO_2_) had substantially positive effect on the improvement of B_2_^L^ quality in B_1_ upgrading in the presence of H_2_, due to the increase of C, H contents, and HHVs and the decrease of N, S, and O contents. The combined Ni-Ru/CeO_2_+H_2_ led to the best B_2_^L^ quality with the highest C+H content, the largest HHV, and the lowest N+S+O content. Overall, the best catalytic effect of Ni-Ru/CeO_2_+H_2_ is likely attributed to positive reactions such as hydrogenation, deoxygenation, denitrogenation, and desulfurization in B_1_ upgrading.

Energy recovery is a key indicator of the effectiveness of a hydrothermal process in capturing the chemical energy of initial algae in the produced biocrude [16]. Due to little variation of biocrude HHVs under different conditions, the energy recovery is largely tracked with the biocrude yield, as displayed in Table 1. It can be noticed that the energy recovery of B_1_ was 62.5% after algae HTL, but after B_1_ upgrading at various conditions, the energy recoveries of B_2_ ranged from 36.9 to 46.2% (average 39.9%) of the heating value of the algae initially loaded into the reactor. Ni-Ru/CeO_2_+H_2_ addition led to the high energy recovery of 46.2% owing to the largest B_2_ yield in this case (see Figure 1). Moreover, the energy recoveries of B_2_^L^ ranged from 23.2 to 32.1% (average 27.9%) of the heating value of the algae loaded initially. Notably, it can be calculated that 49.7–58.6% (average 54.9%) of chemical energy in B_2_ was distributed to B_2_^L^ (i.e., its light biocrude portion). Hence, taking into account the yields, elemental compositions, HHVs, and energy recoveries of both B_2_ and B_2_^L^, Ni-Ru/CeO_2_+H_2_ can be regarded as the optimal additive in all tested cases.

### 3.3. Gas Analysis

Note that Ni-Ru/CeO_2_ is favorable for organic matters gasification to produce a flammable gas mixture in supercritical water [10], so gaseous products are also concerned in this research.  Table 2 provides gaseous products after hydrothermal upgrading of B_1_ at different conditions, so major gases compositions included H_2_, CH_4_, CO_2_, C_2_H_2_, C_2_H_6_, CO, and C_2_H_4_ at the condition in absence of H_2_. Compared with the case without catalyst and H_2_ (i.e., none), the Ni-Ru/CeO_2_ catalyst could significantly improve H_2_ mole fraction (from 29.6% to 41.1%), and meanwhile the postreaction pressure increased from about 2.2 to 3.5 bar. This suggests that Ni-Ru/CeO_2_ led to much more H_2_ and C-containing gases formation during B_1_ upgrading probably mainly via promoting water-gas shift and decarbonation reactions. This outcome is in good accord with the findings that Ru/C [17] and Ni-Ru/CeO_2_ [10] are effective for hydrothermal gasification and the relatively low B_2_ yield in Figure 1 as well. Notably, it reminds us that these flammable gases formed in the water-insoluble biocrude upgrading should be disposed properly in large scale production processes. In the case of H_2_ addition, major identified gaseous products were CH_4_, CO_2_, C_2_H_6_, C_2_H_4_, CO, and unreacted H_2_, but there was no C_2_H_2_ due to hydrogenation effect. Similar gases compositions are also found in catalytic upgrading of duckweed biocrude in subcritical water [18]. The far high H_2_ mole fraction (about 70%) is mainly attributed to initial H_2_ addition (2.0 MPa). Nonetheless, the remarkable reduction of the postreaction pressure in contrast to that before reaction suggests a large amount of H_2_ consumption for heteroatoms removal (likely in the forms of H_2_O, NH_3_, and H_2_S) and hydrogenation in B_1_ upgrading. Certainly, at the same time, there is also H_2_ formation due to biocrude gasification in supercritical water (see the “none” group).

### 3.4. GC-MS Analysis

**Figure 2:**
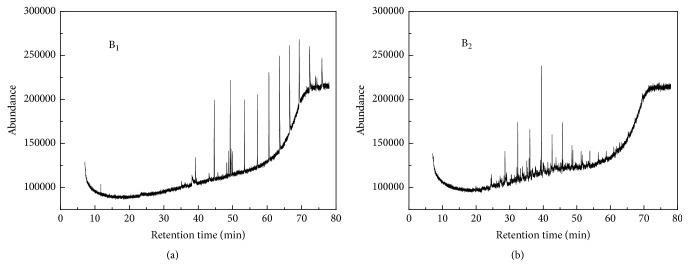
Total ion chromatograms of (a) B_1_ derived from algae HTL at 350°C, 20 min, and (b) B_2_ obtained from B_1_ upgrading at 450°C, 60 min, Ni-Ru/CeO_2_+H_2_ conditions.

Biocrude is produced by complex reactions such as oligomerization, depolymerization, decomposition, and reformation reactions [19–21]. Its compounds compositions are mainly dependent on feedstock feature, since not only lipids but also proteins and carbohydrates are converted into biocrude in algae HTL [22].  Figure 2 exhibits total ion chromatograms of B_1_ and B_2_ derived from B_1_ upgrading at the Ni-Ru/CeO_2_+H_2_ condition. The characteristics of the upgraded water-insoluble biocrude are explored here for the first time. In combination with Table 3 (tentative GC-MS analysis results), it can be confirmed that B_1_ consisted of large amounts of complex compounds such as propanal, 2,2-dimethyl-, oxime; cycloheptasiloxane, tetradecamethyl-; cyclooctasiloxane, hexadecamethyl-; heptadecane; 2-hexadecene, 3,7,11,15-tetramethyl-, [R-[R^*∗*^,R^*∗*^-(E)]]-; 7,8-didehydro-4,5-epoxy-17-methyl-3,6-bis[(trimethylsilyl)oxy]-, (5.alpha.,6.alpha.)-; cycloheptasiloxane, tetradecamethyl-; cyclodecasiloxane, eicosamethyl-; and cyclotrisiloxane, hexamethyl-; 2-methyl-6-(5-methyl-2-thiazolin-2-ylamino)pyridine. Some of them contained N, O, and both them, which is consistent with high N and O contents in this material (see Table 1). Some existing cyclic nitrogenous compounds (e.g., 7,8-didehydro-4,5-epoxy-17-methyl-3,6-bis[(trimethylsilyl)oxy]-, (5.alpha.,6.alpha.)-; 2-methyl-6-(5-methyl-2-thiazolin-2-ylamino)pyridine) are likely produced by Maillard reactions between amino acids and reducing sugars, which are separately formed by the hydrolysis of proteins and carbohydrates [20].

Apparently, the total ion chromatogram of B_2_ looks completely different from that of B_1_, because most compounds appearing in the range of 45–75 min in B_1_ disappeared or reduced. Major compounds in B_2_ appeared in the range of 28–55 min, where the peaks areas sum of all identified compounds took up 95.7% of the total peak area. Main compounds involved pentadecane, tridecane, tetradecane, heptadecane, and dodecane, in which pentadecane and tridecane seem to be the most abundant two species. Thus, hydrothermal upgrading of B_1_ with Ni-Ru/CeO_2_+H_2_ is able to transform high-molecular-weight compounds into low-molecular-weight (i.e., low-boiling-point) compounds, meaning the quality improvement of the upgraded biocrude. More importantly, this catalytic upgrading helps to remove heteroatoms (e.g., O and N) in B_1_ to form the upgraded biocrude with abundant presence of a series of aliphatic saturated hydrocarbons. Reactions such as decarboxylation, dehydroxylation, and deamination contribute to the heteroatoms removal, hydrocarbons formation, and so corresponding increase in carbon content and HHV after hydrothermal upgrading.

### 3.5. NMR Analysis

^1^H-NMR and ^13^C-NMR analysis of biocrude was conducted to identify the types of functional groups. Chemical shift provides information concerning functional group identification, and corresponding peak area can roughly reflect its relative abundance.

#### 3.5.1. ^1^H NMR Analysis

**Figure 3:**
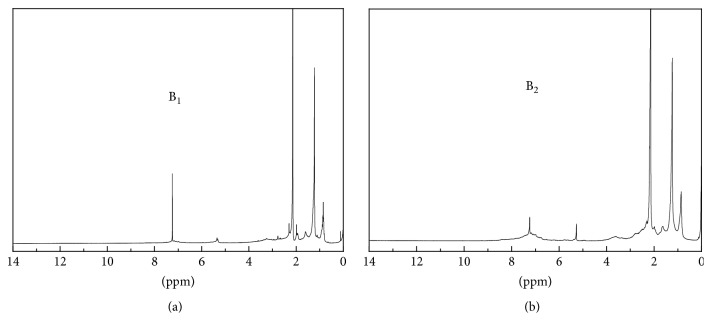
^1^H-NMR spectra of (a) B_1_ obtained from algae HTL at 350°C, 20 min, and (b) B_2_ derived from B_1_ upgrading at 450°C, 60 min, and Ni-Ru/CeO_2_+H_2_ conditions.

Figure 3 illustrates ^1^H NMR spectra of B_1_ and B_2_ derived from B_1_ upgrading at the Ni-Ru/CeO_2_+H_2_ condition. The resonances at 0.8 and 1.2 ppm are characteristics of protons in terminal methyl groups and methylene groups in alkyl chains, respectively [23]. The peak at around 2.1 ppm is consistent with resonance expected from protons on carbon atoms *α* to an acyl group [15]. There was a peak near 7.2 ppm in B_1_ and B_2_, which arises from aromatic protons or conjugated dienes [15, 24], and herein the much smaller peak area in the B_2_ spectrum suggests that the catalytic hydrothermal upgrading by Ni-Ru/CeO_2_+H_2_ can effectively reduce the compounds containing the functional groups above. This is consistent with the findings in the GC-MS analysis. Differently, in the B_2_ spectrum, a clear peak emerged at around 5.2 ppm representing phenolic -OH [24], possibly due to the existence of hydroxyketone in the upgraded biocrude. The appearance of these peaks above reveals the existence of alkyl moieties, carbonyl functionalities, and aromatic or unsaturated molecules in the biocrudes, though in different amounts [9]. Overall, in B_1_ and B_2_ obtained from B_1_ upgrading with Ni-Ru/CeO_2_+H_2_, alkane functionality (0.5–1.5 ppm) and aliphatic *α*-to-heteroatom/unsaturated functionality (1.5–3.0 ppm) [25] were very abundant, due to the presence of alkanes and other compounds with aliphatic methylene and methyl groups.

#### 3.5.2. ^13^C NMR Analysis

**Figure 4:**
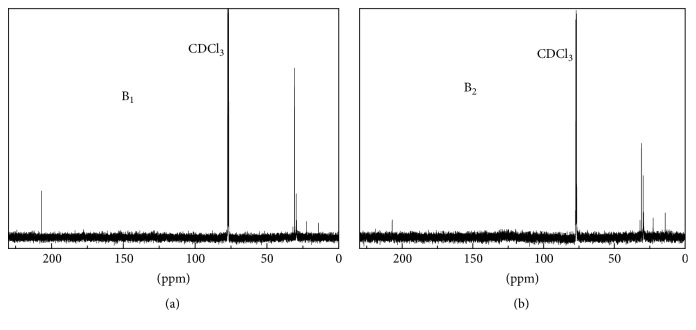
^13^C-NMR spectra of (a) B_1_ derived from algae HTL at 350°C, 20 min, and (b) B_2_ obtained from B_1_ upgrading at 450°C, 60 min, and Ni-Ru/CeO_2_+H_2_ conditions.

Figure 4 displays ^13^C-NMR spectra of B_1_ and B_2_ (at the Ni-Ru/CeO_2_+H_2_ condition). They have larger chemical shift regions and thus provide more details about C-related functional groups. The two spectra possessed some peaks in the 0–55 ppm region, where aliphatic methyl and methylene carbon (such as alkane carbons) atoms appear [4]. This suggests that aliphatic linkage variability lies with chain length or degree of branching within the linkers [24]. In detail, the region above can be divided into 0–28 ppm (short aliphatics) and 28–55 (long and branched aliphatics), and these aliphatic carbon atoms contribute significantly to energy content [25]. Except for the solvent peak at around 77 ppm, there was no signal in the 55–95 ppm region, where carbohydrate carbons appear [25]. Moreover, each biocrude also indicated a peak at about 207 ppm, where carbonyl carbons in ketones and aldehydes appear [8]. The drop of the peak area implies the content reduction of the O-containing compounds above after B_1_ upgrading with Ni-Ru/CeO_2_+H_2_.

### 3.6. FT-IR Analysis

**Figure 5:**
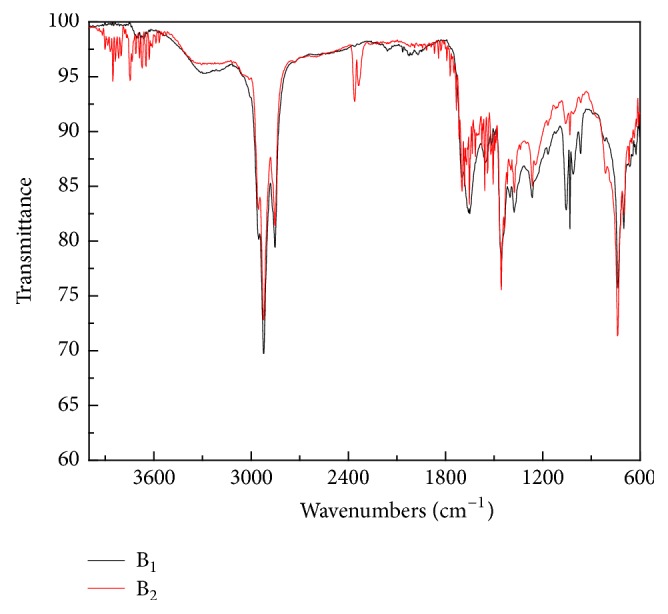
FT-IR spectra of B_1_ derived from algae HTL at 350°C, 20 min, and B_2_ obtained from B_1_ upgrading at 450°C, 60 min, and Ni-Ru/CeO_2_+H_2_ conditions.

Figure 5 elucidates FT-IR spectra of B_1_ from algae HTL and B_2_ obtained from hydrothermal upgrading of B_1_ with Ni-Ru/CeO_2_+H_2_. GC-MS and NMR analysis results show that the two biocrudes contained methylene groups in alkanes, which is proved by further FT-IR analysis herein. Asymmetrical and symmetrical C–H stretching vibrations in aliphatic methylene groups appear in the range of 2800–3000 cm^−1^ [15]. The existence of carbonyl carbon (such as carboxylic acids and esters) is characterized by the bands at 1650–1760 cm^−1^ [8]. The high intensity in these two regions is in accord with a significant amount of hydrogen in the biocrudes being aliphatic [15]. The spectrum of B_2_ also exhibited strong absorbance at approximately 1450 cm^−1^, where the scissoring band in methylene groups emerges [23]. This is probably attributed to some mononuclear aromatic compounds (e.g., naphthalene, 1,2,3,4-tetrahydro-1,1,6-trimethyl- in Table 3) present in the biocrude. In addition, the two biocrudes also had other different functional groups such as methyl, methylene, aromatic, and alkyne groups appearing at ~1378, ~1271, ~1036, and ~735 cm^−1^, respectively, and their different absorbance intensities imply content variations. Different from B_1_, B_2_ presented certain intensity of bands within 2000–2500 cm^−1^ representing triple bonds or cumulative double bonds and large amounts of absorption peaks within 3600–3900 cm^−1^ (e.g., O–H stretching vibrations in hydroxyketone [26]). Hence, hydrothermal upgrading of B_1_ with Ni-Ru/CeO_2_+H_2_ led to obvious variations of functional groups compositions and contents in B_2_.

## 4. Conclusions

In all tested hydrothermal upgrading (at 450°C, 60 min and with added H_2_ and/or catalyst) of B_1_ from algae HTL at 350°C, 20 min, Ni-Ru/CeO_2_ had the best catalytic desulfurization effect and could significantly improve H_2_ mole fraction and rendered much more H_2_ and C-containing gases formation after B_1_ upgrading. Ni-Ru/CeO_2_+H_2_ led to the highest B_2_ yield (27.0 wt%), the best elemental compositions, and the largest HHV (39.94 MJ/kg), as well as the highest B_2_^L^ fraction in B_2_ (57.1 wt%) and the best B_2_^L^ quality. At the Ni-Ru/CeO_2_+H_2_ condition, 46.2% of chemical energy in initial algae could be recovered in B_2_, and average 54.9% of chemical energy in B_2_ was in its light biocrude portion. Ni-Ru/CeO_2_+H_2_ was able to transform high-molecular-weight compounds into low-molecular-weight compounds and led B_2_ to containing a series of abundant aliphatic saturated hydrocarbons such as pentadecane, tridecane, tetradecane, heptadecane, and dodecane. Overall, Ni-Ru/CeO_2_+H_2_ can be regarded as the optimal additive in all tested cases, and there seemingly was synergetic effect between Ni-Ru/CeO_2_ and H_2_ on B_2_ yield and quality improvement after B_1_ upgrading.

## Figures and Tables

**Table 1:** Elemental compositions, HHVs, and energy recoveries of B_1_ (derived from algae HTL at 350°C, 20 min) and B_2_ and B_2_^L^ (obtained from B_1_ upgrading at 450°C, 60 min with different additives).

Additive	Biocrude	Element (wt%)					N/C	O/C	HHV (MJ/kg)	Energy recovery (%)
		C	H	N	S	O				
–	B_1_	77.05	9.76	4.82	0.53	6.84	0.063	0.089	38.81	62.46

None	B_2_	79.74	9.44	4.24	0.37	5.21	0.053	0.065	39.54	38.03
H_2_	B_2_	79.19	9.59	4.37	0.41	5.44	0.055	0.069	39.53	40.61
Ni-Ru/CeO_2_	B_2_	79.63	9.55	4.03	0.19	5.6	0.051	0.070	39.57	36.89
Ni-Ru/CeO_2_+H_2_	B_2_	80.23	9.57	4.12	0.22	4.86	0.051	0.061	39.94	46.24
Ni/CeO_2_+H_2_	B_2_	79.27	8.87	4.55	0.45	5.86	0.057	0.074	38.46	37.80
None	B_2_^L^	80.26	9.49	4.04	0.25	4.96	0.050	0.062	39.82	32.11
H_2_	B_2_^L^	80.63	9.77	3.59	0.35	4.66	0.045	0.058	40.41	30.10
Ni-Ru/CeO_2_	B_2_^L^	81.26	9.57	3.54	0.18	4.45	0.044	0.055	40.35	23.24
Ni-Ru/CeO_2_+H_2_	B_2_^L^	81.9	9.73	3.62	0.19	3.56	0.044	0.043	40.96	29.25
Ni/CeO_2_+H_2_	B_2_^L^	81.4	9.6	3.89	0.16	3.95	0.048	0.049	40.53	24.75

**Table 2:** Gaseous products compositions after B_1_ upgrading at 450°C, 60 min with different additives.

Additive	H_2_ (%)	CO (%)	CH_4_ (%)	CO_2_ (%)	C_2_H_2_ (%)	C_2_H_4_ (%)	C_2_H_6_ (%)	Pressure after reaction (bar)
None	29.6 ± 1.2	5.0 ± 0.8	18.7 ± 2.2	16.1 ± 1.1	12.2 ± 1.8	4.3 ± 0.4	12.5 ± 1.1	2.2 ± 0.2
H_2_	70.7 ± 6.9	2.8 ± 0.4	9.2 ± 2.4	6.6 ± 1.2	–	2.91 ± 0.3	6.5 ± 1.0	9.7 ± 0.4
Ni-Ru/CeO_2_	41.1 ± 2.7	3.2 ± 0.5	16.6 ± 1.9	21.8 ± 1.4	5.7 ± 1.0	2.7 ± 0.2	8.4 ± 1.5	3.5 ± 0.3
Ni-Ru/CeO_2_+H_2_	70.4 ± 7.7	1.1 ± 0.6	9.0 ± 1.7	9.7 ± 1.3	–	1.8 ± 0.3	6.5 ± 0.9	8.8 ± 0.3
Ni/CeO_2_+H_2_	69.8 ± 7.2	2.1 ± 0.4	8.75 ± 1.2	8.9 ± 1.1	–	1.9 ± 0.3	6.2 ± 0.8	8.2 ± 0.4

**Table 3:** Tentative products identifications by GC-MS analysis of B_1_ and B_2_ in Figure 2.

Retention time (min)	Compound	Relative peak area (%)
11.7223^a^	Propanal, 2,2-dimethyl-, oxime	0.3283
38.1796^a^	Benzoic acid, 4-methyl-2-trimethylsilyloxy-, trimethylsilyl ester	1.337
39.2486^a^	Cycloheptasiloxane, tetradecamethyl-	3.2133
43.1274^a^	Diethyl phthalate	0.0338
44.6316^a^	Cyclooctasiloxane, hexadecamethyl-	7.5681
45.6930^a^	Heptadecane	0.6365
48.2967^a^	1-Dodecanol, 3,7,11-trimethyl-	1.3641
48.8617^a^	Hexadecane, 2,6,10,14-tetramethyl-	1.862
49.2741^a^	Cyclononasiloxane, octadecamethyl-	7.8494
49.5718^a^	2-Hexadecene, 3,7,11,15-tetramethyl-, [R-[R^*∗*^,R^*∗*^-(E)]]-	5.1915
57.1387^a^	Cyclodecasiloxane, eicosamethyl-	5.7078
60.5213^a^	Morphinan, 7,8-didehydro-4,5-epoxy-17-methyl-3,6-bis[(trimethylsilyl)oxy]-, (5.alpha.,6.alpha.)-	8.6591
63.6290^a^	Cycloheptasiloxane, tetradecamethyl-	10.5962
66.5381^a^	Cyclodecasiloxane, eicosamethyl-	10.0725
69.3022^a^	Cyclotrisiloxane, hexamethyl-	12.5258
69.7069^a^	Tetrasiloxane, decamethyl-	0.5209
71.5088^a^	Arsenous acid, tris(trimethylsilyl) ester	0.3042
72.3411^a^	(p-Methoxyphenyl)-acetonyl-dimethylsilane	6.9254
72.5015^a^	2-Methyl-6-(5-methyl-2-thiazolin-2-ylamino)pyridine	1.0923
75.8993^a^	2,4,6-Cycloheptatrien-1-one, 3,5-bis-trimethylsilyl-	5.2647
24.5728	Undecane	2.7899
28.6044	Dodecane	6.3133
32.1702	Benzene, 1-(2-butenyl)-2,3-dimethyl-	4.0373
32.4222	Tridecane	10.9529
33.2621	2,4,6(1H,3H,5H)-Pyrimidinetrione, 5-butyl-5-ethyl-1,3-bis(trimethylsilyl)-	3.2033
33.7432	Spiro(9-methylenetricyclo[6.2.1.0(2,7)]undeca-2,4,6-triene)-11,1′-cyclopropane	2.7049
35.1252	Naphthalene, 1,2,3,4-tetrahydro-1,1,6-trimethyl-	3.9456
35.7742	2-Tetradecene, (E)-	3.7452
36.0338	Tetradecane	7.816
39.1873	Cyclododecane	3.3313
39.4240	Pentadecane	17.3115
39.9509	2H-1,2,5-Oxasilaborole, 5-tert-butyl-4-ethyl-2,2,3-trimethyl-	3.3719
42.6309	Hexadecane	4.6292
45.6699	Heptadecane	7.1577
48.5485	Octadecane	5.014
48.8539	Hexadecane, 2,6,10,14-tetramethyl-	4.3843
51.2897	Nonadecane	3.2509
51.5951	3-Butyn-1-ol	2.025
53.8934	Eicosane	2.5267
56.3826	Heptadecane, 2,6,10,15-tetramethyl-	1.4892

^a^The peaks are for Figure 2(a) and other peaks are for Figure 2(b).
